# Encapsulation, Release, and Cytotoxicity of Doxorubicin Loaded in Liposomes, Micelles, and Metal-Organic Frameworks: A Review

**DOI:** 10.3390/pharmaceutics14020254

**Published:** 2022-01-21

**Authors:** Mihad Ibrahim, Waad H. Abuwatfa, Nahid S. Awad, Rana Sabouni, Ghaleb A. Husseini

**Affiliations:** 1 Department of Chemical Engineering, College of Engineering, American University of Sharjah, Sharjah P.O. Box 26666, United Arab Emirates; g00056692@alumni.aus.edu (M.I.); g00062257@alumni.aus.edu (W.H.A.); nawad@aus.edu (N.S.A.); rsabouni@aus.edu (R.S.); 2 Materials Science and Engineering Program, College of Arts and Sciences, American University of Sharjah, Sharjah P.O. Box 26666, United Arab Emirates

**Keywords:** doxorubicin, liposomes, micelles, metal-organic frameworks (MOFs)

## Abstract

Doxorubicin (DOX) is one of the most widely used anthracycline anticancer drugs due to its high efficacy and evident antitumoral activity on several cancer types. However, its effective utilization is hindered by the adverse side effects associated with its administration, the detriment to the patients’ quality of life, and general toxicity to healthy fast-dividing cells. Thus, delivering DOX to the tumor site encapsulated inside nanocarrier-based systems is an area of research that has garnered colossal interest in targeted medicine. Nanoparticles can be used as vehicles for the localized delivery and release of DOX, decreasing the effects on neighboring healthy cells and providing more control over the drug’s release and distribution. This review presents an overview of DOX-based nanocarrier delivery systems, covering loading methods, release rate, and the cytotoxicity of liposomal, micellar, and metal organic frameworks (MOFs) platforms.

## 1. Introduction

Cancer refers to uncontrolled cell division due to DNA mutations. It occurs when cells are induced to over-proliferate, caused by the permanent activation of proto-oncogenes in upregulated oncogenes [1]. Throughout their normal life cycle, cells divide controllably, differentiate, and eventually die through the programmed cell death mechanism known as apoptosis. However, mutations perturbing the ordinary growth pathway of the cells can introduce behavioral changes on genetic and epigenetic levels, where these transformed cells exhibit different growth characteristics, including enhanced mobility and the decreased contact inhibition of growth mechanisms [2]. Some cancer cells incur tumor-initiating abilities and can travel to other parts of the body through the bloodstream or lymph vessels (metastasis) [3].

Anticancer drugs are classified into three categories: chemotherapy, immunotherapy, and hormonal therapy. These therapies can target tumor cells at the DNA, RNA, or protein level [4,5,6]. Chemotherapy is the invasive anticancer therapeutic regimen used worldwide. It was introduced by the German chemist Paul Ehrlich and has been used to treat cancer since the beginning of the 20th century [7,8,9].

The formulations of antineoplastic agents were divided, according to their chemical structure and mechanism of action, into alkylating agents, antibiotics, antimetabolites, topoisomerase inhibitors, and others [5,6,7,10,11,12]. The last decades have witnessed colossal efforts to discover novel chemotherapeutic drugs including, but not limited to, doxorubicin, epirubicin, pirarubicin, methotrexate, pemetrexed, 5-fluorouracil, paclitaxel, cisplatin, gemcitabine, and others [3,10,13]. Chemotherapeutics are usually administered either orally or intravenously to achieve systemic distribution, thus maximizing their effect. Unfortunately, the practicality of chemotherapy drugs is counteracted by their lack of selectivity, causing grave damage to healthy cells with high division rates like bone marrow, hair follicles, and gastrointestinal epithelia. In addition, both spermatogenesis and oogenesis processes are highly susceptible to the cytotoxic side effects of chemotherapeutics.

Although surgery and radiotherapy are ideal for treating localized solid cancers, their successful use is hampered in many cases where cancer has metastasized to other organs in the body. Thus, chemotherapy is commonly offered to patients at advanced cancer stages. It is a systemic therapy that targets rapidly dividing cells by employing agents that interfere with their life cycle and replication mechanisms. It is advantageous over conventional therapies because it can act throughout the whole body, thus targeting cancer cells that metastatically spread far from the primary tumor site and helping to shrink bulky tumors that might otherwise be unresectable [14]. From the 1960s onwards, the success of chemotherapeutic drugs in treating various advanced cancers led to the development of the so-called adjuvant therapy, in which chemotherapeutics are combined with surgery to eradicate potential cancer dissemination or recurrence [15,16]. Sometimes, chemotherapy combined with radiotherapy is more effective than either modality alone.

**Figure 1:**
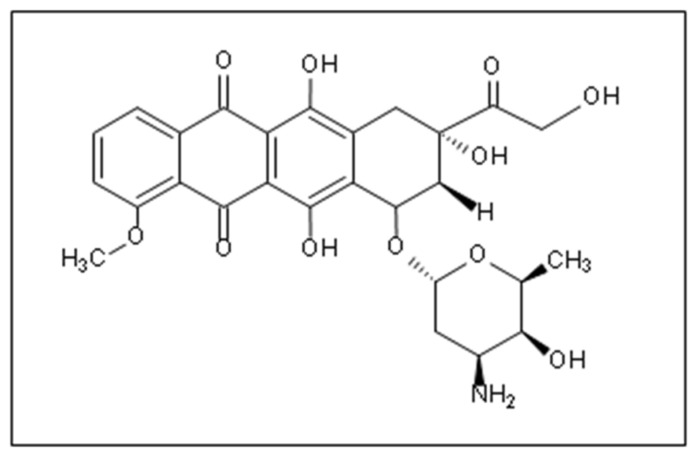
The chemical structure of DOX [7].

Among the most widely used class of chemotherapeutics are anthracycline agents, which are prevalently successful in achieving short-time cancer progression inhibition, high response, and improved survival rates [17]. Anthracyclines are the first anti-tumor antibiotics approved by the Food and Drug Administration (FDA), among which is Doxorubicin (DOX), one of the first effective anthracycline antibiotic cytotoxic drugs discovered. It was isolated from the red pigment-producing soil bacterium; Streptomyces peucetius via mutagenic treatment [18]. DOX (Figure 1) is extensively used to treat lymphomas, leukemia, as well as ovarian, breast, small cell lung, stomach, and liver cancers [4,7,19,20].

**Figure 2:**
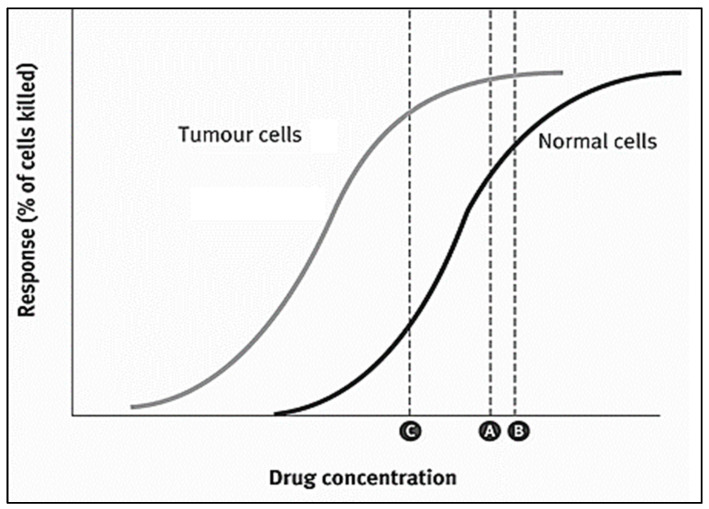
Dose responsive curve for chemotherapy drugs kills both tumor cells and healthy cells. The therapeutic dose (**A**) is close to the toxic dose (**B**). It is not safe to give this drug at a therapeutic dose. A safe dose (**C**) is chosen for administration to patients. Reproduced with permission from [14], Elsevier, 2008.

Like most anthracycline antibiotics, DOX oxidizes and inhibits the activity of topoisomerase II, an enzyme responsible for DNA transcription and replication, generating unstable, highly active free radicals that damage the DNA and cause cell death [4,7,19]. Although it is an effective anti-tumor agent, its side effects are most evident on cells exhibiting high division rates, such as hair follicles and the gastrointestinal tract lining; thus, hair loss, digestive tract ulcerations, vomiting, nausea, and diarrhea are all common complications/side effects [21]. Also, it has been known to stimulate myelosuppression and induce cardiotoxicity by the upregulation of apoptosis receptors in cardiomyocytes [22,23]. Clinical investigations by Von Hoff et al. [24] showed 7.5% cardiomyopathy occurrences associated with cumulative DOX doses of 550 mg/m^2^. Thus, cumulative doses of 400–500 mg/m^2^ are generally employed for free administration once every three weeks to prevent cardiotoxic effects. In addition, DOX is a vesicant and can cause blistering and necrosis if extravasation occurs at the time of drug administration [14]. Moreover, drug resistance in the tumor cells is another problem that limits the clinical use of DOX [19,25,26]. In general, the toxicity of chemotherapeutic drugs limits their effective dosage threshold. In most cases, a therapeutic dose of the chemotherapy drugs capable of efficiently killing the tumor cells is highly toxic to the fast-growing normal cell and, therefore, cannot be given to patients (Figure 2). Instead, a lower dosage capable of destroying considerable numbers of tumor cells with lower toxic effects on normal cells is utilized [14].

Following the treatment with chemotherapy drugs, tumor cells show slower replication times compared to healthy cells, which can regenerate at controlled replication rates. This led to the development of the intermittent administration of chemotherapy which allowed the total regeneration of normal tissues but not that of tumor tissues, leading to reducing but not eliminating the toxicity of these drugs [27]. Chemotherapeutics are associated with other challenges that hinder their use, including:

(a) Limited solubility in aqueous solutions: most of the chemotherapy drugs are hydrophobic. Thus, solvents are used to solubilize these drugs, which increases their toxicity.
(b) Poor specific targeting of the cancer cells, i.e., high toxic dosages, are delivered to healthy as well as cancer cells.
(c) Cancer cells can develop resistance to chemotherapy drugs, a phenomenon known as multi-drug resistance (MDR). This results in minimal cell death and the expansion of drug-resistant tumors.

## 2. Nanoparticles as Drug Delivery Systems (DDS)

Due to the detriment to the patients’ quality of life and the potential lethality of some of the side effects associated with conventional chemotherapy, novel drug delivery systems (DDS) aim to reduce the adverse side effects and enhance the specificity of chemotherapeutic drugs. In 1964, Cheng initiated the use of encapsulation of enzymes and other biologically active materials in semipermeable vesicles and tested their use to suppress the growth of lymphosarcoma in a mice model [28,29]. This approach has evolved and been extensively extended into nanomedicine, biotherapeutics, blood substitutes, drug delivery, enzyme/gene therapy, cancer therapy, nanoparticles, liposomes, bioencapsulation, regenerative medicine, nanobiotechnology, and nanotechnology [30]. Earlier studies on the transformed cancer cells showed that they had adopted many biochemical strategies to support their uncontrolled growth [31,32]. A deep understanding of the key enzymes and antagonistic pathways of synthesis and catabolism involved in tumor progression resulted in the development of “targeted therapy”. Targeted DDS are nanoplatforms that incorporate nanoparticles (NPs) as drug delivery vehicles, ranging in size from 1 nm to 1000 nm [33,34]. These include, but are not limited to, gold NPs, dendrimers, polymeric nanogels, micelles, metal-organic frameworks (MOFs), liposomes, and quantum dots. The synthesis routes of these NPs vary and are generally divided into chemical and biological ways, where the latter are preferred as they are safer and innocuous [35].

**Figure 3:**
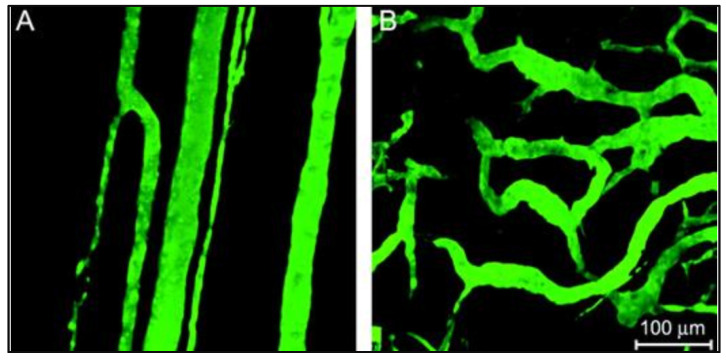
(**A**) a normal vascular network where the vessels are parallel-aligned next to each other; (**B**) a tumoral vasculature with chaotic defective arrangement. Adapted with permission from [37], Oxford University Press, 2006.

Utilizing these nanocarriers for the remote delivery of appropriate dosages to targeted anatomical sites under controlled release conditions can overcome the shortcomings of traditional treatment approaches. The localized accumulation of the nanoparticles at the neoplastic site is mainly achieved by passive and/or active targeting routes. In passive targeting, the intrinsic features of the tumor neovasculature beneficially provide fenestrations where the NPs accumulate. As a tumor undergoes rapid, chaotic growth, it exhibits a disorganized vascular network and becomes hypoxic due to insufficient oxygen supply. Tumor cells can secrete growth factors to induce vascularization to resolve this hypoxia and get nutrients from neighboring healthy cells by a process referred to as angiogenesis [35,36]. The newly formed capillitial endothelium in tumor tissues is disorganized and leaky with improper lymphatic drainage and inadequate transport phenomena mechanisms (Figure 3) [37]. Consequently, the tumor site suffers from abnormal molecular and fluid dynamics. These features allow the NPs to extravasate into the tumor’s interstitium [38] and accumulate inside the diseased tissues, in a phenomenon referred to as the enhanced permeability and retention (EPR) effect.

Yet, designing DDS with complete dependence on passive targeting has significant limitations, such as the possible accumulation of the NPs in the spleen and liver as these organs have fenestrated vasculature as well as the inability of the NPs to sufficiently penetrate deep enough through the complex tumoral network due to heterogeneities in structure [39]. Thus, the development of systems that incorporate both passive and active targeting mechanisms became imperative. Since tumor cells overexpress receptors that participate in growth and survival pathways, such receptors make promising active targets. To this end, nanocarriers could be conjugated to natural ligands to target these receptors leading to their accumulation and internalization by the cancer cells [40].

This review will discuss three types of nanocarriers: liposomes, micelles, and metal-organic frameworks (MOFs) delivering the antineoplastic agent DOX to treat different types of solid cancers, including their DOX loading techniques, encapsulation efficiency, and their ability to eradicate tumors.

## 3. DOX Delivery Systems Based on Liposomes

**Figure 4:**
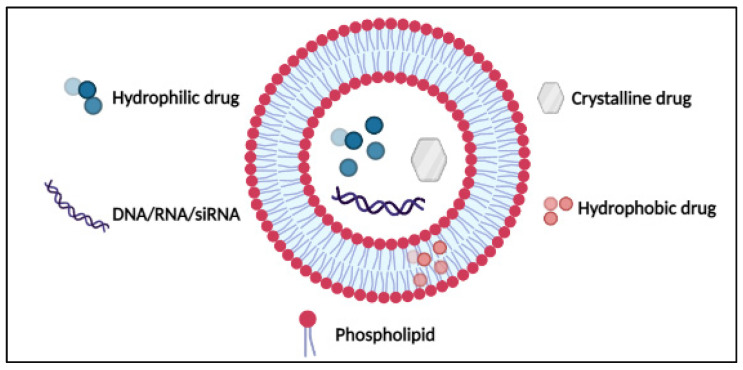
Structure of liposomes. Reproduced from [46], IntechOpen, 2014.

Bangham and co-workers used multilamellar bilayer lipid microspheres and liposomes as models of biological membranes for basic research in the 1960s [41,42]. It was Gregoriadis’ group that first used liposomes as drug carriers in the 1970s [43]. Later, the multilamellar onion-like microspheres were developed into submicron dimension bilayer lipid vesicles (Figure 4). The hydrophilic heads of the phospholipids are directed outwards, whereas the hydrophobic tails are directed inwards. Cholesterol is usually added to the phospholipids to increase the mechanical rigidity of liposomes [44,45]. Liposomes have gained immense interest as drug carriers because of their biocompatible, biodegradable, non-toxic, and targetable nature. Moreover, they deliver a high drug-to-lipid ratio and can also be functionalized to avoid rapid elimination from the body [46].

**Figure 5:**
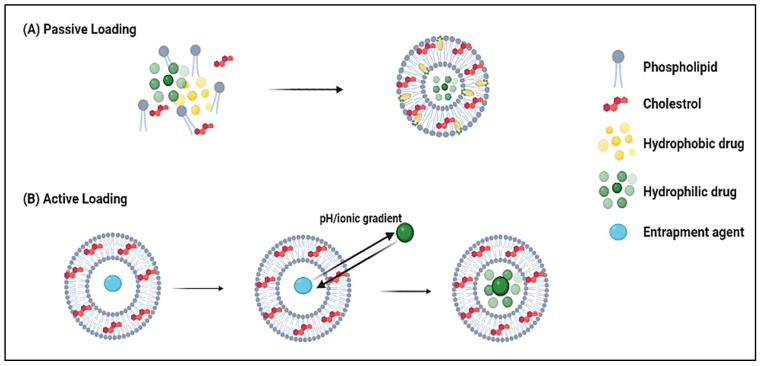
Two approaches for DOX loading into liposomes: passive and active. (**A**) before “pasive” and (**B**) before “active”.

For the encapsulation of lipophilic DOX into liposomes, two loading routes (i.e., passive and active) are generally used (Figure 5). One of the most common passive loading methods is based on the thin-film hydration method, where the drug solution is added during the liposome formation process, either while the film is being formed or during the hydration step. To enhance trapping efficiency, the drug is typically added during the film formation of liposomes containing negatively charged (acidic) lipids [44,45]. The dried lipids are initially randomly orientated, but upon exposure to an aqueous solution, the water causes the molecules to self-organize into a bilayer configuration to minimize entropic interactions. In the presence of hydrophilic and/or hydrophobic drugs in solution, some of the drug molecules would passively partition and accumulate in the cores and/or the shells of the liposomes. Although this method is simple, it yields very small drug encapsulation percentages. In 1983, DOX was passively entrapped in the bi-layer of positively (phosphatidylcholine-cholesterol-stearyl amine) and negatively (phosphatidylcholine-cholesterol-phosphatidylserine) charged liposomes. The drug was added to the phospholipids before film formation [47]. One day after DOX loading, the mean diameters of positive and negative non-filtered multilamellar liposomes were between 500-600 nm and 1 μm, respectively. The maximum loading capacities ranged between 60-75 mmol DOX/mol phospholipid for the negative and approximately 55 mmol DOX/mol phospholipid for the positive, non-filtered, non-sonicated liposomes [47].

On the other hand, the size stability and release profiles were determined over an extended period. The filtered and sonicated/centrifuged negatively-charged liposomes were stable during the entire storage period and maintained average diameters of 270 and 120 nm, respectively. However, the sonicated/centrifuged positively-charged liposomes were not stable and had a size of 120 nm with a standard deviation of 40 nm over 2 weeks of storage. The negative filtered liposomes released only 10% of DOX during 75 days, whereas 40% was released over 20 days from the negative sonicated/centrifuged liposomes. For the release from positive sonicated/centrifuged liposomes, the baseline was set after 12 days, and the cumulative release was ~30% after 48 days [47].

Other passive loading methods include the freezing-and-thawing technique (FAT). The liposomal solution undergoes a series of freezing and thawing processes, causing the interlayer distance within the liposomes to increase [48]. As a result, ice crystals cause transient holes and pores to form within the liposomal structures, allowing for passive drug entrapment. However, this method yields 5–20% loading efficiency and requires heavy post-processing to remove the excess drug. Likewise, the reverse-evaporation method can load drugs with efficiencies up to 50%. Still, the formulations lack reliable in vivo drug retention and often suffer from rapid cargo release under physiological conditions. Thus, occurrences of “burst release”, where a large fraction of the entrapped drug is abruptly released due to poor association and retention in the vesicles, combined with the relatively low drug-to-lipid entrapment ratio, typically do not exceed 0.05% (*w/w*), making it imperative to divert to other loading techniques [48]. Moreover, the high variability and dependence of the encapsulation efficiency on operational factors like the drug’s solubility, nanocarriers’ chemistry, size, and preparation method deemed active loading methods more preferable and more commonly used for the stable entrapment of DOX into liposomes.

**Figure 6:**
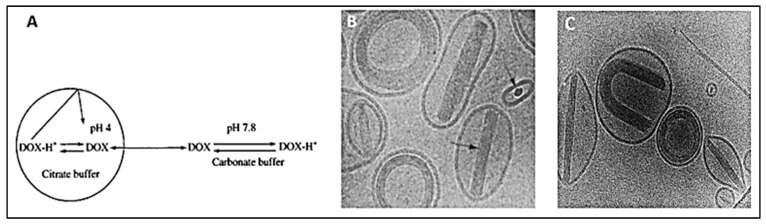
(**A**) DOX loading via citrate transmembrane gradient method, (**B**,**C**) confocal images of DOX-loaded liposomes showing the coffee-beans liposomes appearance where DOX-citrate complexes appear in rod, circular and U-shaped structures. **A**,**B** are adapted with permission from [50], Elsevier, 2001. C is adapted with permission from [51], Elsevier, 1998.

On the other hand, active methods, also known as remote loading methods, employ pH gradients between the internal acidic core buffer of blank liposomes and the external buffer containing DOX at neutral physiological conditions. Under such conditions, the drug molecules incubated with the formed liposomes can permeate selectively through the lipids transmembrane, where they become protonated intra-vesicularly [45]. Work by Bally and colleagues [49] investigated the effects of creating a membrane potential by altering the pH of the intraliposomal solution from a 300 mM citrate buffer at pH 4.0 to a HEPES buffer at pH 7.4 on the entrapment of DOX and biogenic amines in liposomes. High encapsulation efficiencies of up to 98% were achieved, with a drug-to-lipid ratio of 0.3 (*w/w*%) corresponding to 400 nmol DOX/µmol phospholipid (~260 mM internal concentration). Also, retention times (T_50_) significantly increased from 1 h to 30 h in passively versus actively DOX-loaded formulations, respectively. The proposed “citrate” method resulted in highly stable liposomes with remarkably enhanced drug retention and release characteristics, with the added benefit of cost-effectiveness. A commercially available formulation of liposomal DOX, Myocet^®^, is loaded using the same approach (Figure 6). The Myocet^®^ liposomes (~150 nm) are made from egg phosphatidylcholine and cholesterol at a ratio of 55/45 (mole/mole %). As a result of the pH gradient, DOX molecules diffuse and accumulate in the liposomal cores and form a complex with the citrate anions. The drug entrapment efficiencies (>95%) and drug-to-lipid ratio (~0.27) exceeded the theoretical predictions as the bundled fibers complexes allowed higher DOX loading beyond its solution solubility limits. The DOX-citrate complexes resembled the appearance of coffee-bean liposomes, where the linear bundles prevented drug leakage and premature release. The formulation also exhibited prolonged circulation times and improved in vivo tissue distribution compared to free DOX [50]. According to Li et al. [51], 99% of DOX loaded into citrate buffered liposomes appears in the form of fiber aggregates. The threshold for forming these fibrous-like DOX-citrate structures can be as low as ~20 mM internal DOX concentration. As the DOX concentration increases, the fibers begin cross-linking via citrates and then pack into bundles. Kanter and co-workers [52] demonstrated that the Myocet liposomal formulation effectively reduced the effects of myocardial degeneration, commonly associated with anthracycline-based treatments. Histological analysis on beagle dogs treated with conventional DOX versus those treated with Myocet (single i.v. administration every 3 weeks; 8 cycles; cumulative dose = 12 mg/kg) showed that the latter group did not suffer from any anthracycline-induced cardiotoxicity, while the first group had lesions of myocardial degeneration.

Along the same lines, Haran et al. [53] generated a pH gradient across the liposomes using ammonium sulfate salt. The liposomes initially encapsulated a 300 mM ammonium sulfate solution at pH 5.5 in a pH 7.4 external buffer. The higher concentration of ammonium ions on the inside led to the diffusion of the neutral ammonia molecules, and with every molecule diffusing from the core, a proton was left behind. Due to salting-out effects and the acidification of the intraliposomal compartment, high fractions of DOX can accumulate in the liposomes in an aggregated form. Liposomes loaded using this technique exhibit a prolonged stable storage period beyond 6 months because of the gelation effects of DOX with the sulfate salt, which inhibit membrane re-permeation. Alyane et al. [54] examined the stability and release behavior of liposomes loaded with DOX via the ammonium sulfate transmembrane method. Liposomes comprised of hydrogenated egg yolk phosphatidylcholine (HEPS), cholesterol, and DSPE-PEG2000 at a molar ratio of 185:1:15 showed an encapsulation efficiency exceeding 90%, with a drug-to-lipid ratio of 1:20 (*w/w*). Upon incubation at 37 °C for 24 h in culture media and PBS, the liposomes retained 98% and 90% of the encapsulated drug, respectively, showing minimal leakage under the stated conditions. When the incubation medium was changed to phosphate buffer at pH 5.3, the maximum release achieved was 37%. The pH responsiveness of the liposomes suggested their suitability for controlled release in acidic compartments (i.e., tumor tissues) as they would keep their cargo intact under physiological conditions. The study also revealed that encapsulating DOX in the liposomes decreased the agent’s uptake by rat myocardial H9C2 cells, suggesting the decreased cardiac toxicity of the formulation. Flow cytometry and MTT assay analysis showed that the toxic effects of DOX were reduced when compared against that of free DOX, up to 20 h of incubation time. After 20 h, the liposomal formulation became more toxic to the cells than the free one [54].

DOXIL^®^ is a commercially available PEGylated liposomal DOX formulation that uses the ammonium sulfate gradient method to encapsulate the drug. An in vivo study by Sakakibara et al. [55] investigated the performance of DOXIL^®^, conventional DOX-liposomes, and free DOX on the treatment of human lung tumors. The DOXIL^®^ liposomes were composed of hydrogenated soy phosphatidylcholine, cholesterol, PEG-DSPE, and dl-a-tocopherol in a molar ratio of 56.1:38.2:5.5:0.2. In contrast, the non-PEGylated liposomes had phosphatidylglycerol, phosphatidylcholine, cholesterol, and DL- α -tocopherol in a molar ratio of 1:4:3:0.02. To assess and compare the different treatment groups (dosage: 1.5 mg/kg) of anti-tumor activity, human lung tumor xenografts were engrafted into the gonadal fat pad of severe combined immunodeficient (SCID) mice. The tumor eventually metastasized from the primary site into the mice’s peritoneal cavity (i.e., liver, lung). It was concluded that the PEGylated formulation successfully suppressed the primary tumor growth and arrested metastasis in the peritoneal cavity, while the free DOX was only able to stop the growth of the primary tumor without significant effects on preventing the spread. The PEGylated liposomes showed 5-folds higher circulation times than conventional liposomes, while the uptake by vital organs like the spleen was reduced. Also, a significant increase in extravasation and accumulation of the PEGylated formulation was observed, as considerable amounts were detectable at the tumors after 1 week following administration [55]. Another study [56] compared the in vivo pharmacokinetic performance of free DOX with PEGylated and non-PEGylated liposomal formulations. Interestingly, the PEGylated liposomal DOX clearance rate decreased by 100-folds (Cl = 0.023 L/h), and its half-life (t1/2 = 83.7 h) was prolonged by 8-folds, compared to free DOX (Cl = 25.3 L/h, t1/2 = 10.4 h). Moreover, the distribution volume decreased significantly from 364 L to 139 L to 3.0 L in the free DOX, non-PEGylated, and PEGylated liposomal DOX, respectively. This conclusion demonstrated that PEGylation prevents premature drug release and that most of it remains entrapped without leakage.

Fritze et al. [57] introduced another DOX remote loading method based on a phosphate ((NH_4_)_2_HPO_4_) transmembrane gradient, using different ammonium and sodium salts. The liposomes were composed of egg phosphatidylcholine (EPC) and cholesterol in the molar ratio 7:3. After hydrating the liposomes with 300 mM salt solutions at neutral pH, they were incubated with DOX.HCl for 12 h at 7 °C to achieve a drug-to-lipids ratio of 1:3 (mol/mol). The sizes of liposomes containing DOX dissolved in different ammonium and sodium salts, as well as their encapsulation efficiencies (EE%), are summarized in Table 1. Results showed that loading did not significantly alter the size of the liposomes, and those loaded with the ammonium salts gradients showed higher EE than the sodium salts. Synergistic effects are suggested when using ammonium salts as they act as a reservoir for donating free protons when DOX is internalized and protonated in the acidic interior of the liposomes. Thus, intraliposomal protonation and DOX precipitation led to increased encapsulation efficiencies. Further analysis was conducted to assess the impact of varying the intravesicular ammonium concentration on liposome size and EE. DOX was dissolved in 10 mM isotonic HEPES buffered saline (HBS), 50, 100, 200, and 300 mM ammonium phosphate at pH 7.2. The sizes of the DOX-loaded liposomes were found to be 84 ± 0.4, 102 ± 1.4, 114 ± 3.6, 95 ± 1.5, and 92 ± 1.6 nm, respectively, with EE of 2.81, 23.52, 61.03, 83.35, 97.98%. Increasing the ammonium ion concentration augmented the effects of drug protonation and intravesicular acidification, which led to increased DOX diffusion across the transmembrane gradient. The least efficiency (<5%) was observed when incubating in HBS, which contains no phosphate ions (no decrease in the intraliposomal pH level), while the highest efficiency approaching 100% was observed at the highest concentration of the ammonium phosphate (300 mM).

Another study [58] investigated the effect of varying the bilayer composition of DOX entrapment and release efficiency. Three liposomal formulations were tested, which included non-thermosensitive (NTS) liposomes composed of L-α-phosphatidylcholine (PC), thermosensitive (TS) liposomes composed of dipalmitoylphosphatidylcholine (DPPC) and distearoylphosphatidylcholine (DSPC), lyso-thermosensitive (LTS) liposomes composed of DPPC, DSPC, and 1-palmitoyl-2-lyso-glycero-3-phosphocholine (P-lyso-PC). All three formulations contained small amounts of cholesterol. The encapsulation efficiency of DOX dissolved in 0.9% NaCl solution was very low for TS and LTS liposomes (5.8 and 5.6%), while it was slightly higher (17.29%) for NTS liposomes because of the possibility of controlling the temperature effectively during the loading (above the phase transition temperature). In vitro drug release was modeled at controlled hyperthermia conditions (41–42 °C), and results showed that for a period of 5 h, 2%, 36%, and 54% DOX diffused from NTS, TS, and LTS liposomes. The relatively lower encapsulation efficiency and higher release performance of LTSL resort to the temperature-induced membrane instability when operating at temperatures beyond the lipids transition temperature [58]. Generally, drug loading via active loading depends on several factors besides the drug’s physiochemical properties, like the extraliposomal medium condition, pH, conductivity, electrolytic activity, loading duration, as well as operating temperatures [44].

Research efforts have also focused on the synergistic effects of using release triggering modalities, like ultrasound and pH, on the internalization and efficacy of DOX. Pitt et al. [59] studied the in vivo performance of ultrasonically-triggered DOX-loaded liposomes on BDIX rats bearing colonic carcinoma. The liposomes were comprised of soy phosphatidyl choline, cholesterol, DSPE-PEG, and alpha-tocopherol combined in the molar ratio 3:1:1:0.004, and DOX was entrapped via the ammonium sulfate gradient method. Using sonication remarkably enhanced the DOX release kinetics and effects on mice. The group treated with liposomes followed by ultrasound exposure (20 kHz for 15 min, once for 4 weeks) showed significant regression in tumors growth to an immeasurable size by the end of the treatment period. Likewise, another in vivo study [60] on mice bearing SCC7 murine squamous carcinoma cells showed that sonication using pulsed high-frequency US (HFUS) enhanced the performance of the proposed liposomal drug delivery system. Fluorescent spectrophotometry results proved that the mean DOX concentration in the sonicated tumors was 124% more than in the control, which received the liposomal treatment but without sonication. Work by Zhang et al. [61] synthesized pH-sensitive liposomes by modifying their surfaces via the insertion of poly(2-ethyl-2-oxazoline)-cholesteryl methyl carbonate (PEOZ-CHMC) copolymer. The performance of these liposomes was compared to others grafted with PEG-DSPE chains. DOX hydrochloride was loaded into both types of liposomes by the ammonium sulfate transmembrane gradient method. It was revealed that functionalization with PEOZ-CHMC and PEG-DSPE had no effects either on the drug’s encapsulation efficiency, which was 97.3 ± 1.4, or on the size, which was ~120 nm. In vitro release done via dialysis showed that the release profile from PEOZ-CHMC-DOX liposomes in PBS at pH 5.0 significantly surpassed that observed at pH 7.4, suggesting a strong relationship between medium acidity and cargo release from the modified liposomes. Moreover, MTT assay results proved a direct relationship between the antiproliferative effects of the PEOZ-CHMC-DOX liposomes and pH conditions. The pH-sensitive formulation showed higher activity inhibition to the MCF-7 cells at lower pH; herein, pH can be used as a triggering mechanism for drug release.

As mentioned, scheming highly efficient liposomal drug delivery systems with complete dependence on passive targeting is hampered by limitations like the possible accumulation of the nanocarriers in the spleen and liver as these organs have fenestrated vasculature and their incapability to sufficiently penetrate deep enough through the complex tumoral network due to heterogeneities in structure [62]. Benefiting from the tumor cells’ overexpression of receptors to aid in augmenting their growth and survival pathways, such receptors make promising active targets. To this end, nanocarriers can be conjugated to the ligands complementing these receptors to enhance DOX accumulation and internalization by the cancerous cells. Xing and co-workers [63] examined the in vitro effects of functionalizing DOX-loaded PEGylated liposomes with a DNA aptamer (Apt-Urn liposomes) on MCF-7 cells. Flow cytometry results showed a 6.6-fold increase in drug uptake and efficacy of the functionalized loaded liposomes as opposed to the conventional ones, as the fluorescence response upon 4 h of incubation with the treatments corresponded to 93.6% and 57.0%, in the cells treated with Apt-Urn and control liposomes, respectively. Moreover, MTT assay results analyzed the cytotoxic effects of the Apt-Urn liposomes on cell viability. The cells were treated with different liposomal concentrations followed by an incubation period of 6 h, then re-cultured in fresh media, and the MTT test was done after 72 h. At a liposomal DOX concentration of 500 nM, cells treated with the functionalized liposomes exhibited a viability of 57.0 ± 6%, whereas cells treated with the control liposomes exhibited a viability of 92.4 ± 9%, evidencing the superior localization and internalization of the nanocarriers as a function of surface modification.

Yang [64] conducted a recent preclinical investigation on DOX-loaded liposomes composed of EPC and cholesterol for treating head and neck cancer. The study compared three treatment groups: free DOX, DOX-loaded liposomes, and DOX-loaded peptide-conjugated liposomes. Results showed the dependence of the encapsulation efficiency on the cholesterol content in the formulations, as it increased from 20% to 79% upon the incorporation of cholesterol with EPC. Varying the EPC and cholesterol ratio yielded different entrapment efficiencies ranging from ~48% to ~82%. Both liposomal formulations, the functionalized and the conventional ones, showed sustained release profiles over a period of 40 h, but the DOX-loaded peptide-conjugated liposomes had a faster in vitro release behavior. In vivo analysis of the formulations’ efficacy was carried out on nude mice bearing HSCC (human squamous cell carcinomas) xenograft. The tumor progression was suppressed when treated with both liposomal formulations; however, the functionalized liposomes arrested metastasis and increased the median survival time of the animals in that group by 100%. The enhanced performance was due to the increased accumulation at the tumor site, resorted to localized targeting by binding to the overexpressed surface-specific receptors (Hsp47/CBP2) abundant on the head and neck cancer cells.

Another study [65] examined the performance of DOX-loaded liposomes, functionalized with the monoclonal antibody Trastuzumab (TRA) for the enhanced targeting of breast cancer cells overexpressing the Human Epidermal growth factor Receptor 2 (HER2). The liposomes consisted of cholesterol, DPPC, and DSPE-PEG(2000)-NH_2_ at a molar ratio of 30:65:5, respectively. DOX was loaded via the ammonium sulfate transmembrane gradient method, yielding a drug-to-lipids ratio of 1:6. The cargo release from the liposomal formulations, conventional and TRA-conjugated, was triggered using pulsed ultrasound. In vitro results showed synergistic effects on the DOX uptake and internalization upon sonication and surface modification with TRA. Similarly, Chowdhury and co-workers [66] designed an in vitro study where they tested different targeted (functionalized with Aptamer-A6) and untargeted liposomal-DOX formulations for the localized treatment of HER2-overexpressing breast cancers. Twelve liposomal formulations (F1-F12) were prepared using the thin-film hydration method by varying the compositions of the phospholipid mixtures. The liposome sizes slightly increased upon DOX loading and ranged from 98.7 to 181.2 nm, with 74.9 to 94.1% entrapment efficiencies. The optimized formulations for further complexation with Aptamer-A6 were determined based on the smallest size to benefit from the EPR effect and the highest encapsulation efficiency. Formulations 5 and 8 (10 mg, 150 mg, 40 mg, 40 mg, 20 mg, and 0.25 mg of DOX, POPC, DOTAP, DOPE, DSPE-mPEG2000, and Mal-PEG, respectively) had sizes of 101.70 ± 14.04 nm and 98.7 ± 13.25 nm, respectively, and DOX entrapment efficiency of 92.8% and 94.1%, respectively. These results suggest the statistical insignificance of the hydrophobicity and charge (i.e., cationic) of the used phospholipids on the encapsulation efficiencies. However, the presence of PEG chains and the charge of the liposomal complexes directly affect their stability and integrity in long-term storage. Formulations with cationic lipids exhibited a relatively smaller size and extended shelf-life lasting up to 10 weeks, and PEG prevented liposomes from cross-linking and aggregating. Integrating Mal-PEG into the formulations aided in linking the Aptamer-A6 at its amino-terminal. In vitro analysis was done on SKBR3 and MCF7 cells, which overexpress HER2 on their surfaces. It was observed that F5 was internalized the fastest and the most by both cell lines, as flow cytometry uptake results showed the fluorescence intensities upon 2-h incubation to be 98.6% and 66.5%, respectively. SKBR3 cells uptake of F5 compared to MDA-MB-231 cells (HER2 negative cell line) was more by 1.79 times, substantiating the merit of targeted therapy. Table 2 presents some studies involving liposomal DOX.

**Figure 7:**
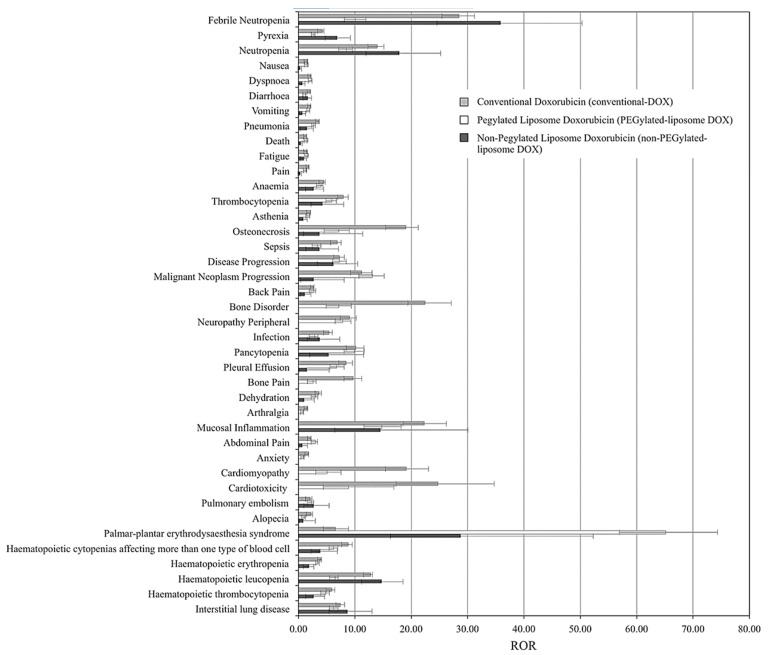
Summary of ROR of conventional, PEGylated, and non-PEGylated liposomal DOX in the FAERS database. (High resolution image at https://doi.org/10.1371/journal.pone.0185654.g002 accessed on 3 January 2022). Adapted from [75], PLOS, 2017.

As many liposomal DOX formulations have successfully translated to clinical applications, and others are still in the clinical testing phase, it is essential to compare their performance and overall pharmacovigilance against the free drug. Table 3 presents a comparison between phase III clinical trials results of two current commercially available liposomal DOX formulations, namely Myocet^®^ and DOXIL^®^, against conventional DOX. Clinical studies have shown that DOXIL^®^ reduced the constraints on the dose limits of free DOX administration, in addition to reducing the cardiotoxic and myocardial damage incidences even at high doses exceeding the 500 mg/m^2^ threshold [73,74]. Fukuda et al. [75] presented the first large-scale study that compared the adverse effects (AEs) associated with treatments of conventional, PEGylated (DOXIL^®^, Caelyx^®^, and LipoDox^®^), and non-PEGylated (Myocet^®^) liposomal DOX formulations, based on data collected from 7,561,254 patient reports (from 2004 to 2015) retrieved from the Food and Drug Administration Adverse Event Reporting System (FAERS). The analysis was based on the top 30 AEs correlated with conventional DOX treatments, including nausea, diarrhea, vomiting, anemia, cardiomyopathy, and cardiotoxicity. Figure 7 summarizes the findings, which show the reporting odds ratios (RORs) of each treatment for each AE. ROR is a pharmacovigilance index that reflects the chances of occurrence, detection, and prevention of AEs of drug formulations. The lower the ROR, the more patient-friendly the formulation is. As reported, all DOX treatments have been correlated with severities of myelosuppression, cardiotoxicity, alopecia, nausea, and vomiting; however, both liposomal DOX formulations show relatively better safety profiles than conventional DOX.

Nonetheless, PEGylated liposomal DOX had higher ROR for palmar-planar erythrodysesthesia (PPE), stomatitis, and mucositis than the non-PEGylated and conventional formulations, though a complete understanding of these AE’s pathophysiology has not yet been fully elucidated. A meta-analysis study was previously conducted by Rafiyath et al. [76], which covered randomized controlled trials of treatments of different tumors with conventional and liposomal DOX. The investigation analyzed data of 2220 patients in total, of which 1108 and 1112 were treated with conventional and liposomal (PEGylated and non-PEGylated) DOX, respectively. The results are generally aligned, evidencing that the liposomal formulations show better therapeutic indications, but careful consideration should be given to treating patients suffering from PPE with PEGylated DOX. In terms of pharmacokinetics and biodistribution performance of conventional, non-PEGylated, and PEGylated liposomal DOX, a preclinical evaluation by Tomkinson et al. [77] reported that the area under the plasma concentration-time curve (AUC; μg hr/mL) was 4, 45, and 900, respectively, and the elimination half-lives (hr) were 0.2, 2.5 and 55, respectively. Although the prolonged circulation times of PEGylated formulations are preferable in terms of therapeutic manifestations, several clinical trials [78,79] have associated that with increasing the risk factors of PPE. The intricacy of correlating PEGylated liposomal DOX toxicities with dose and pharmacokinetic parameters between pre- and clinical settings calls for population pharmacokinetics guidance tools [77] to improve patient outcomes and the overall quality, safety, and efficacy of the liposomal treatments [80]. 

## 4. DOX Delivery Systems Based on Micelles

Micelles are amphiphilic colloidal block copolymers resulting from the self-assembly of molecules. They are divided into two categories, i.e., low-molecular-weight surfactant micelles and polymeric micelles. DOX was conjugated effectively to the terminal hydroxyl group of PLGA fond in PLGA-b-PEG di-block copolymer micelles via a chemical reaction and entrapped in the same micelle physically with sizes of 61.48 ± 7.17 and 58.21 ± 6.21 nm, respectively. The loading efficiency and capacity of chemically conjugated DOX were calculated to be 99.09 ± 1.81% and 2.18 ± 0.04, respectively. They were higher compared to only 23.18 ± 3.18% loading efficiency and 0.51 ± 0.07% capacity for physically loaded DOX. The release experiments were performed in phosphate-buffered saline (PBS) at 37 °C. Approximately 45% of chemically conjugated DOX was released during the first day. The entire amount was delivered after 4 days, while physically entrapped DOX took longer to release only 50% of DOX after 2 weeks. In vitro cytotoxicity of free DOX and DOX-PLGA-b-PEG against HepG2 cells was examined. The loaded drug (IC_50_ = inhibitory concentration to produce 50% cell death = 0.02 μg/mL) was found to be more toxic than the free drug (IC_50_ = 0.3 μg/mL) [84].

A new pH-sensitive DOX-micelles delivery system was proposed by Bae and co-workers [85]. Mixed micelles were prepared from poly(His-co-Phe (16 mole%))-b-PEG (80 wt.%) blended with PLLA-b-PEG (20 wt.%), denoted as PHSM (20%), or folated PLLA-b-PEG (20 wt.%), denoted as PHSM(20%)-f. The size of mixed micelles at neutral pH was 110 nm and reduced slightly at pH 6.5 and 6. On the contrary, it increased dramatically to reach 900 nm at pH 5.5. This occurred due to the dissociation of PLLA-b-PEG from the mixed micelles due to incompatibility with ionized poly(His-co-Phe). The drug encapsulation was carried out via dialysis, and the loading efficiency was 85% with 20 wt.% drug loading content (DLC). The DOX release rate from PHSM (20%) was determined at different pH environments under mechanical shaking (100 rev/min) at 37 °C. As expected, the slowest release was observed at neutral pH and started accelerating in acidic pH. After 10 h, approximately 25, 38, 75, and 85% were released at pH 7.4, 6.5, 6, and 5.5, respectively. The complexity of polymerization, purification, and characterization of mixed micelles limits their use.

In vitro cytotoxicity experiments were conducted for free DOX, folated, and non-folated mixed micelles against ovarian A2780 wild-type cells with a drug concentration of 1 μg/mL. The free DOX showed lower cell viability (between 17–20%) compared to high viability percentages in the case of loading the DOX in PHSM(20%) without the folate moiety at pH of 7.4, 7, 6.5, and 5.5, which reached up to approximately 80, 85, 75, and 30%, respectively, whereas the same cell viability percentage of 18% was achieved at pH 5.5 of free and loaded drug. The difference in the cell viability of folated micelles-free drug was insignificant at all pH values. Another cell viability test was performed against a DOX-resistant cell line (A2780/DOXR). Free DOX and DOX/m-PHSM (20%) exhibited very high cell viability with percentages greater than 85%. Contrary to that, DOX/m-PHSM(20%)-f showed around 15, 19, 21, 32, 50% at pH of 7.4, 7, 6.5, and 5.5, respectively. It was concluded that conjugating active targeting folate molecules to the mixed micelles make them more effective in killing ovarian A2780 wild-type and A2780/DOXR cell lines [85].

Hsieh et al. [86,87] reported loading neutralized DOX-HCL in triblock PEG-PCL-PEG (EC220E) copolymers under mixing for 3 h. The empty and loaded micelles sizes were 85 ± 2 and 92 ± 7 nm, respectively. The loading content was 7.4%, with an efficiency of 49%. An in vitro release study was performed at pH 7.4 and 5.4 at 37 °C. During the first 6 h, burst release with percentages of 20 and 25% at pH 7.4 and 5.4, respectively, was observed (due to the diffusion of DOX located within the hydrophilic shell or near the surface of micelles). Then, the accumulated drug delivery rate became relatively slow to reach approximately 27% and 37% at pH 7.4 and 5.4, respectively, after 48 h. Finally, the cytotoxicity of these treatments was evaluated using two human breast cancer cell lines. The IC_50_ values of treating MCF-7 using free and encapsulated DOX were 0.031 and 0.218 μg/mL, respectively. When DOX-resistant cells (MCF-7/adr) were treated, both free (IC_50_ = 4.68 μg/mL) and loaded DOX (IC_50_ = 5.96 μg/mL) showed somewhat similar half-maximal inhibitory concentrations.

Moreover, DOX hydrochloride was loaded in one of the most frequently used amphiphilic tri-block copolymers in drug delivery applications, namely Pluronic^®^F127, grafted with 5% and 10% O-succinyl chitosan [88]. The loading was carried out by mixing the drug in 5, 7, and 10% (*w/v*) grafted micelles solutions at 250 rpm for 12 h in the dark. The mean diameters of loaded micelles ranged between 34-40 nm. The encapsulation efficiencies were calculated to be 73.69 ± 0.53 and 74.65 ± 0.44%. DOX release mechanism was studied at 37 °C in PBS at pH 7.5. During the first 24 h, burst release was observed with 39–42% and 29–39% delivery for 5% and 10% nanoparticles. Then, the remaining amount was released slowly to reach 85–90% and 73–86% after 22 days. From the in vitro experiments against MCF-7 cells, it was found that the DOX loaded nanocarriers (IC_50_ ranged from 0.19 to 0.42 μg/mL) were more cytotoxic than Free DOX (IC_50_ = 0.67 μg/mL), while the empty nanoparticles showed minimal toxic effects (IC_50_ ranged from 4.32 to 7.60 mg/mL) [88].

DOX was co-encapsulated with an optical fluorescence imaging agent, CdSe quantum dots (QDs), in theranostic phospholipid-based micelles prepared to DSPE-PEG and DSPC [89]. The average size of QDs-DOX micelles formulation was around 50 nm. The release profile of 46 μg/mL loaded DOX from the QDs-DOX micelles was investigated by incubating the QD-Dox micelles with 1% aqueous tween 80 (mimics the in vivo serum conditions) at 37 °C. Almost 50% of the DOX was released after 100 h, while the cumulative release percentage reached 95% after seven days. Incubating free DOX and QD-Dox micelles with HeLa cells for 24 h resulted in more than 65 and 50% cell survival, respectively, at a drug concentration of 5 μg/mL [89].

Furthermore, 3-helix micelles were utilized to load DOX using the thin-film hydration method [90]. The resulting micelles were loaded with 8 wt.% DOX and were 15 nm in size. The release was investigated in PBS and in the presence of serum albumin (mimics the conditions after intravenous injections) at 37 °C and found to be slow, as only 11 and 12% of DOX were recovered after 20 h, respectively. The results of in vitro cytotoxicity of free DOX and loaded 3-helix micelles against PPC-1 (human line) and 4T1 (syngenic mouse line) showed comparable cell viabilities. After 36 h incubation with PPC-1 at 5 μg/mL drug concentration, both free and encapsulated DOX showed similar low cell viability. However, the cytotoxic effect of micellar DOX was slightly higher compared to the free drug after 72 h of incubation. At low drug concentrations (1 μg/mL), free DOX was more effective in killing the cells than micellar DOX. The incubation of 5 μg/mlL of the free drug and DOX loaded in 3-helix micelles with 4T1 cells for 36 h resulted in low cell viability, while all the cells were killed after 72 h of incubation. On the other hand, using the low drug concentration of 1 μg/mL showed relatively high cell viability with more cytotoxic effects of free DOX compared to the loaded DOX following 72 h of incubation [90].

Cross-linked (CL) and non-crosslinked (NCL) PEG-P(LL14- LA14), PEG-P(LL18-CCA4/LA14), and PEG-P(LL18-CCA8/LA10) block micelles were used to load and release DOX [91]. The sizes of NCL PEG-P(LL14- LA14) were 56.5, 77.6, and 87.5 nm with theoretical DLC of 9.1, 16.7, and 23.1 wt.%. DOX-loaded cross-linked copolymers were slightly smaller with sizes of 55.7, 66.9, and 78.2 nm and lower DLC of 6.6, 11.8, and 15 wt.%, respectively. The loaded NCL PEG-P(LL18-CCA4/LA14) micelles had sizes of 44.9, 125.3, and 199.5 nm with theoretical DLC of 9.1, 16.7, and 23.1 wt.%, respectively. These loading contents were higher than those of the loaded CL micelles with sizes of 44.7, 94.2, 144.1 nm with DLC of 7.7, 13.3, and 15.9 wt.%, respectively. NCL and CL DOX-PEG-P(LL18-CCA8/LA10) had sizes of 88.3 and 78.7 nm, respectively. The theoretical DLC of non-crosslinked PEG-P(LL18-CCA8/LA10) was calculated to be 9.1 wt.%, while it was 6.4 wt.% for the cross-linked micelles [91].

Drug release kinetics from 20 μg/mL micelles were investigated at 37 °C under shaking (200 rpm) [91]. At neutral pH, CL PEG-P(LL14- LA14) and PEG-P(LL18-CCA4/LA14) released only 20% of the drug in 24 h. In comparison, the corresponding NCL micelles released 57.2 and 68.3%, respectively of the encapsulated DOX under the same conditions. In an acidic environment (pH 5.0), the release from CL PEG-P(LL18-CCA4/LA14) was triggered to reach 30% (probably due to cleavage of the acid amide bonds between PLL and CCA), whereas it was not affected significantly in the case of PEG-P(LL14- LA14). In the presence of 10 mM glutathione (GSH), the release from CL PEG-P(LL18-CCA4/LA14) increased notably to reach 86.0% and 96.7% at pH 7.4 and 5.0, respectively, after 24 h. On the other hand, 89.4% and 79.5% of DOX were released from CL PEG-P(LL14-LA14) copolymers under the same reductive conditions at pH 7.4 and 5.0, respectively. The addition of cis-1,2-cyclohexanedicarboxylic acid (CCA) resulted in higher loading and faster release. After a 48-h treatment of HeLa cells using CL PEG-P(LL18 CCA4/LA14) and CL PEG-P(LL14-LA14), the IC_50_ values were 12.7 and 21.2 μg/mL, respectively. Additionally, the incubation of HepG2 with the same cross-linked micelles for 48 h resulted in 12.4 and 20.9 μg/mL, respectively. However, free DOX showed more effective cytotoxicity for both cell lines compared to DOX loaded in CL micelles [91].

**Figure 8:**
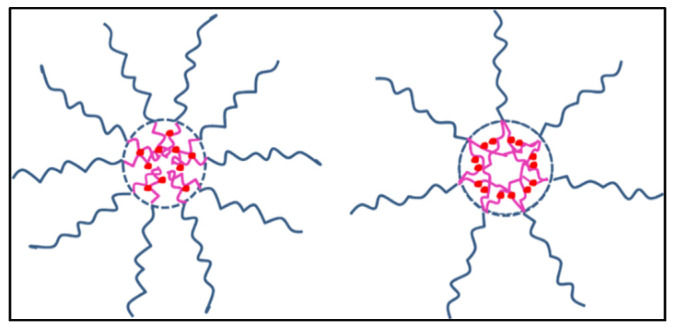
Linear (left) and Y-shaped DOX loaded copolymers (right) Adapted from [92]. MDPI, 2014.

Another DOX-micelles DDS was reported by Sui et al. [92]. They encapsulated the drug in two linear [PEG-P(Glu)62 and PEG-P(Glu)62] and two Y-shaped copolymers [PEG-P(Glu26)2 and PEG-P(Glu31)2] via an acid-labile hydrazone linker using the dialysis method against PBS (pH 7.4, 10 mM) and deionized (DI) water. Figure 8 represents the structure of the loaded copolymers. The sizes of loaded PEG-P(Glu)26, PEG-P(Glu)62, PEG-P(Glu26)2, and PEG-P(Glu31)2 were measured to be 149.9 ± 3.7, 231.4 ± 8.3, 141.3 ± 5.2, and 165.6 ± 2.4 nm, with DLC of 9.92 ± 0.25, 18.8 ± 0.18, 16.2 ± 0.12, and 18.2 ± 0.45%, respectively. The release patterns were examined at 5 and 7.4 pH values. Approximately 70% of DOX was released in 72 h from the Y-shaped micelles, which was higher than only 55% released from the linear micelles. All micelles showed similar release at neutral pH, with less than 45% of the drug being released. To evaluate the cytotoxicity of blank PEG-P(Glu)26 and PEG-P(Glu26)2, HeLa cells were incubated with the polymeric micelles for 24 h. They were not very toxic at a concentration up to 50 μg/mL. On the other hand, the IC_50_ values after 72 h incubation of DOX-conjugated PEG-P(Glu)26 and PEG-P(Glu26)2 with HeLa cells were 0.063, 0.517, and 0.673 μM, respectively. In other words, PEG-P(Glu26)2-DOX were more toxic than PEG-P(Glu)26-DOX at the same concentrations. Table 4 summarizes some preclinical and clinical studies on micellar-based DOX DDS, discussed in [93].

## 5. DOX Delivery Systems Based on Metal-Organic Frameworks (MOFs) 

**Figure 9:**
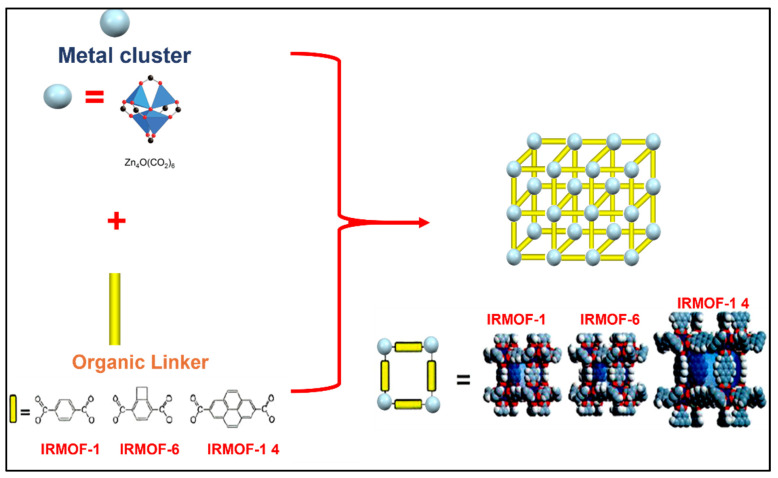
Metal-organic frameworks structures.

Recently, Metal-organic frameworks (MOFs) have attracted great interest among scientists due to their unique physical and chemical properties (Figure 9). MOFs are a new class of hybrid porous crystalline materials, known as coordination polymers, consisting of metal clusters or metal ions connected by organic linkers to create one-, two- or three-dimensional networks [94]. The flexible combination of organic-inorganic units distinguishes MOFs from traditional porous materials and has enabled scientists to develop thousands of new MOFs since its first discovery in 1989 [95,96].

For the past few decades, MOFs have been investigated and reported for several applications including, water purification [97,98], separation [99,100], gas storage [101,102], catalysis [103,104], sensing [105,106] and energy [107,108]. On the other hand, MOFs have gained an increasing attention in the biomedical field such as in imaging [109,110,111], drug delivery [112,113,114,115] and biological sensing [116,117] owing to their superior properties including high surface area (i.e., ∼7000 m^2^/g) [118], biocompatibility [119], wide range of pore size (i.e., 2–50 nm) [120], tunable frameworks high porosity and low density (0.2 to 1 g/cm^3^) [121,122].

In particular, MOFs as drug delivery systems offer several advantages, for instance: (1) high encapsulation capacity due to their ultrahigh porosity; (2) toxicity can be controlled by choosing biocompatible metals and organic linkers [123,124]; (3) targeted delivery can be achieved by alternating the surface structure of the MOFs through surface modification using stimuli-responsive molecule or preforming post-synthetic surface modification [125,126].

Horcajada et al. [127] succeeded in encapsulating DOX in MIL-100(Fe) nanoparticles. They achieved 8.5 wt.% loading capacity after 24 h of incubation, and this percentage increased slightly to reach 9.1 wt.% when the impregnation was repeated. The release mechanism was studied at 37 °C in PBS under agitation. For 9.1 wt.% DOX payload, almost half of the entrapped drug was released during the first day, while the remaining amount was delivered at a very slow pace to complete after 13.5 days [127,128]. The IC_50_ values for MIL-100(Fe) nanoparticles incubated with HeLa cells and J774 for 24 h were evaluated as 1100 ± 150, 700 ± 20 μg/mL, respectively [129].

Another example of the DOX-MOF delivery system was developed by Chakraborty and co-workers using ZIF-7 and ZIF-8 [130]. DOX solution was added to the two ZIFs, and the mixture was stirred for 48 h. The drug encapsulation percentages were 40% and 52% inside ZIF-7 and ZIF-8, respectively. The release mechanisms of the loaded ZIFs were investigated under different conditions. First, they were measured at different pH ranges (7.4, 6.0, and 5.0) in a time interval ranging from 0 to 2.5 h. The reason behind studying the release at acid environment resides in the acidity of tumor microenvironments. ZIF-7 did not show any release in the neutral and acidic environments, while ZIF-8 showed an insignificant increment of fluorescence intensity at pH 7.4, and it started increasing to reach 4 times and 6 times above its initial value at pH 6.0 and 5.0, respectively [130]. As expected, ZIF-8 showed an efficient pH-sensitive DDS [131].

Additionally, the release behavior was observed by contacting the ZIFs with a biomimetic membrane. Upon contact of ZIF-8 with 30 mM SDS micelles (above critical micelle concentration), the fluorescence intensity increased by 1.5 times within 1.5 h. When DMPC liposomes were used, the release increased slightly (the fluorescence intensity increment was only 1.1 times its initial value). In contrast, the initial intensity increased by approximately 1.5 times in the presence of DMPC-DMPG (9:1) liposomes. The contact of ZIF-7 particles with SDS micelles showed almost 1.5 times increment in the fluorescence intensity within a period of 3 h. Compared to ZIF-8, ZIF-7 showed relatively less intensity increment when it contacted DMPC/DMPG (9:1) liposomes [130]. After a 24-h incubation of ZIF-8 particles with J774 and HeLa cells, the IC_50_ values for MIL-100(Fe) nanoparticles incubated with HeLa cells and J774 for 24 h were 25 ± 1.0 and 100 ± 10 μg/mL, respectively [129].

Zheng and co-workers further proposed a novel method, called one-pot synthesis, to load DOX in ZIF-8 during MOF preparation [132]. The encapsulation of DOX molecules was carried out by mixing a DOX solution with the metal and organic precursors for 15 min, and the maximum loading capacity obtained was found to be 20 wt.%. The loaded particles, DOX@ZIF-8, did not show any release under neutral pH, while they released the drug for 7–9 days at pH 5.0-6.0 [132]. The IC_50_ values for MIL-100(Fe) nanoparticles incubated with HeLa cells and J774 for 24 h reached 1100 ± 150, 700 ± 20 μg/mL, respectively [129]. Mechanically downsized gadolinium(III)-based MOF (MG-Gd-pDBI) was also used to encapsulate DOX [133]. After stirring 0.33 mg/mL DOX solution with MOF particles for 24 h, the loading capacity was calculated to be 5.0 wt.%, whereas it reached 12 wt.% when the DOX concentration was increased to 2.0 mg/mL. Nearly 44% of the drug were released from 5 wt.% DOX loaded MG-Gd-pDBI at pH 5.0 after 5 days. In contrast, the release was much slower (22%) at neutral pH, and the entire release was achieved after 15 days. The IC_50_ value of 5 wt.% DOX loaded MG-Gd-pDBI incubated with U 937 cells for 48 h was evaluated to be 75 μg/mL [133].

Recently, a unique DOX delivery system based on UMCM-1-NH–Py gated by carboxylatopillar[5]arene (CP5) has been designed [134]. The loading experiment was conducted in two steps. First, UMCM-1-NH–Py particles were suspended in DOX hydrochloride-PBS mixture for 12 h. Then, the capping agent (CP5) was added, and the whole mixture was stirred for 48 h. At neutral pH, the loaded capped MOF did not release the DOX. However, the release started increasing upon lowering the pH to 2.0 and reached 55% after 7.5 h. Finally, in vitro cell viability was carried out for UMCM-1-NH-Py and CP5-capped UMCM-1-NH-Py after incubation with normal human embryonic kidney (HEK) 293 cells for 24 h at different concentrations. Both MOFs (capped and uncapped) showed low cytotoxicity as the cell viabilities were still high (approximately 55% and 70% for UMCM-1-NH-Py and CP5-capped UMCM-1-NH-Py, respectively) at a high concentration (50 μg/mL) [134].

Novel MOF-based Fe_3_O_4_@UiO-66 core-shell composites were used successfully to incorporate DOX [135]. Different concentrations of DOX dissolved in PBS at pH 8.0 were stirred with the magnetic composites for 24 h, and the drug payload was evaluated to be 2.5 and 66.3 wt.% when the amount of DOX increased from 0.15 to 15 mg, respectively. DOX release kinetics were studied in PBS at pH 4.0, 5.0, 6.0, and 7.4 under shaking. The release percentages at pH 4.0 and 5.0 were measured to be about 36.1% and 21.6%, respectively, whereas only 17.1% and 13.8% of encapsulated DOX were released at the higher pH values (6.0 and 7.4) for 41 days. In vitro experiments were conducted for Fe_3_O_4_@UiO-66, free and loaded DOX on HeLa cells. The empty MOFs did not show any cytotoxicity after 24 h of incubation, even at a high concentration (500 mg/L). On the other hand, both free and loaded DOX killed about 60% of the cells at a low concentration (20 mg/L) [135]. Table 5 presents a few DDSs based on DOX-loaded MOFs to treat different cancers.

Even with the current research advances in MOF-based chemotherapeutic platforms, the move from bench to bedside is hampered by several potent obstacles [136,137]. These challenges include (1) difficulties in scaling up the production, (2) incompatibility with biosafety measures, (3) the need for careful identification of targeting biomarkers and effective conjugation, and (4) FDA regulations compliance.

## 6. Concluding Remarks and Future Directions

Nanocarriers such as liposomes, micelles, and MOFs present diverse and interesting designs of promising drug delivery systems that have been rapidly evolving over the years and continue to be improved/upgraded to achieve their potentials. Liposomes are one of the most explored and successful nanocarriers with many liposomal formulations at the clinical pharmaceutical preparation stages. To date, there are more than eighteen liposomal formulations already approved by the FDA to treat cancer and other diseases [142]. Compared to the liposomes, micelles and MOFs are synthesized from artificial materials and, therefore, are more susceptible to elimination by the RES, affecting the amount of these nanocarriers accumulating in the tumor. The main advantage of liposomes is their high versatility, which allows them to successfully carry out different functions. This is due to the ability to manipulate their designs by controlling the type of natural or synthetic phospholipids used in their preparation using different headgroups (different charge) and diverse fatty acids chains with different lengths and saturation levels (different transition temperatures). In addition, a wide range of compounds can be added to the phospholipid bilayer, such as PEG, cholesterol and targeting ligands such as carbohydrates, peptides, proteins, and antibodies.

However, some issues still need to be addressed to improve the stability of liposomal formulations, such as lipid oxidation and aggregation. In addition, the large-scale production of liposomes is a complex and challenging process. It requires a high level of precision due to the need to constantly test each produced batch to ensure the purity and reproducibility of the used technique. Enhancing the liposomes with PEG (pegylated or stealth liposomes) improves their circulation time and their accumulation inside solid tumors while considerably reducing the cardiotoxicity of DOX. However, clinical trials have shown that pegylated liposomes are associated with new side effects such as skin toxicity and mucositis. Although those side effects are less severe than those caused by doxorubicin treatment, it is important to improve the design of the pegylated liposomes to eliminate their toxicity.

Micelles have a smaller size than liposomes and MOFs, making it easier for them to benefit from the EPR effect when delivering DOX to tumors. In addition, micelles’ ability to self-assemble and protect their encapsulated drug is a unique feature that allowed these macromolecules to be investigated as nanocarriers capable of delivering DOX as well as small particles like proteins and genes. Their simple assembly makes them highly feasible for large-scale production compared to liposomes and MOFs. However, their low stability is a major disadvantage that needs to be addressed. When micelles are injected into the bloodstream and dilute, the concentration decreases below the critical micelle concentration. As a result, these nanovehicles may disassociate, releasing their encapsulated drug before reaching the targeted site. More understanding of the in vivo behavior of micelles should be the focus of future studies. So far, limited studies have investigated the correlation between micelle stability/in vitro drug release and drug pharmacokinetics. Generally, the main two obstacles to the clinical success of micelles are their stability issues and the lack of specific characterization tools. While several structural designs and improved preparation methods are being developed, these improvements mustn’t complicate the simple structure of the micelles, which may make their large-scale production a complex process and technically challenging. The progress to clinical translation depends on securing a robust manufacturing process that is cost-effective and meets the regulatory requirement, especially with complex micelles.

MOFs have interesting advantages over liposomes and micelles as they have the largest surface area/volume ratios and have a highly porous structure. Thus, MOFs can load more DOX and deliver higher local concentrations of DOX compared to micelles and liposomes. However, MOFs are still behind the two other nanocarriers clinically. Understanding the in vivo toxicity of MOFs, drug kinetics while loading and releasing, the mechanism of MOFs degradation, and pharmacokinetics are still the main focus of current research. Overcoming these limitations is essential to fully realizing the promising potential of those unique nanocarriers as DDS in clinical applications. MOFs’ success in clinical trials will also depend on understanding the mechanism of their cellular uptake by the cancer cells.

Liposomes, micelles, and MOFs are injected into the blood and expected to reach the tumor site while still intact, and DOX is encapsulated inside them. Therefore, all three types of nanocarriers are faced with similar challenges; the physical force applied on them by the circulating blood and their elimination by the reticuloendothelial system (RES) as well as the renal system. Another major challenge is the heterogeneity of the EPR effect between the different solid tumors and within the same tumors. The tumor microenvironment is complex and comes with unique conditions and different extracellular matrix (ECM) compared to the normal cells, which directly affect DOX delivery to the cells. Adding a targeting molecule will surely enhance the active targeting ability of these nanocarriers and their binding/uptake by the cancer cells once they reach their target. However, it will not improve their chances of reaching the tumor while facing the many challenges associated with their blood circulation. It is impossible to determine how much of the nanocarriers, targeted or non-targeted, will reach the tumor and at what concentration. Scientific efforts should be directed not only to enhance the properties of the nanocarriers but also to understanding tumor vasculature. Efforts aiming to regulate vessel permeability and the ability to physically disturb the vessels surrounding tumors, e.g., a pre-treatment using photo-immunotherapy with antibody-photosensitizer conjugate, which has shown a 24-fold increase in nanocarriers’ accumulation at the tumor site [143], are all essential to allow these nanocarriers to benefit fully from the EPR effect.

The use of nanocarriers, including those discussed in this review, to deliver DOX to solid tumors is an excellent and efficient method to deliver this important and prevalent anti-neoplastic agent to the targeted sites while reducing its side effects. There is no ideal drug delivery nanocarrier. Each DDS showed advantages as well as disadvantages. In addition, some of these systems were successful and made it to clinical trials, while others were commercialized. Yet, research efforts continue to improve and enhance the current DDS to unlock the potentials of nanocarriers. Future clinical trials must increase the number of participants to produce robust results regarding the relationship between the produced side effects, the effect of the patient’s age, and the treatment regime. One of the possible future turning points in their application is the use of specially designed nanocarriers for the personalized treatment of cancer patients depending on the tumor characteristics and patients’ conditions.

## Figures and Tables

**Table 1:** Summary of the size and encapsulation efficiency of loaded liposomes achieved via different salts.

Salt Gradient	Size ± SD (nm)	EE (%)
Ammonium Phosphate	129.3 ± 3.7	98
Ammonium Sulfate	129.2 ± 2.9	95
Ammonium Acetate	115.9 ± 1.0	77
Ammonium Citrate	114.9 ± 1.2	100
Sodium Phosphate	113.4 ± 1.6	52
Sodium Sulfate	111.8 ± 1.9	44
Sodium Acetate	113.4 ± 1.6	16
Sodium Citrate	151.7 ± 3.8	54

**Table 2:** Different liposomal-based DDS encapsulating DOX via the (NH_4_)_2_SO_4_ transmembrane gradient method.

Preparation Method	Target Cancer	Functionalization	Study Model	Triggering Modality	Findings	Ref.
Ethanol injection	osteosarcoma	Estrogen	In vitro flow cytometry and MTT analysis on MG63 (estrogen overexpressing) cells and LO2 (negative liver cells). Ex vivo imaging of MG63 tumors extracted from Male BALB/c nude mice.	Redox-sensitivity and glutathione responsiveness	Loaded decorated liposomes size~110 nm. Exhibited high encapsulation efficiency. *Ex vivo* analysis of the functionalized liposomes showed more selective accumulation in tumor tissues compared to other vital organs, and in vitro results showed higher cytotoxicity towards overexpressing cells.	[67]
Thin-film hydration	Lymphoma	anti-CD19 moiety; PEG grafted by disulfide links (mPEG-S-S-DSPE)	In vitro MTT assay In vivo model: Female BALB/c Cr Alt B/M mice bearing Namalwa cells	pH sensitivity	Liposomes decorated with cleavable PEG chains rapidly dissociated in the plasma. The pH-sensitive liposomes, targeting the CD19 epitope excessively abundant on B-lymphoma cells, showed increased selective cytotoxicity towards these cells, and enhanced release kinetics at lower pH levels.	[68]
Post-insertion; mixing with preformed DOXIL	Cancer Stem Cells (CSCs)	anti-CD44 monoclonal antibody (mAb)	In vitro flow cytometry and MTT assay on C-26 and NIH-3T3 (non-tumor) cells. In vivo model: female BALB/c mice bearing C-26 colon carcinoma.	N/A	Functionalization of DOXIL liposomes significantly increased their size. The IC_50_ values were lower on the C-26 cell line overexpressing CD44, while higher values were reported for the negative cell line (NIH-3T3).	[69]
Solvent evaporation	Various cancers	Cationic Polymethacrylate Eudragit RL100	In vitro flow cytometry and MTT assay on MCF7/adr and H22 cells. In vivo model: ICR mice bearing aggressive liver cancer H22 cells.	N/A	Functionalization of liposomes with Polymethacrylate derivatives increases their cellular internalization and antitumoral activity. The in vivo results showed that four injections of the functionalized formulation led to tumor size reduction by 60%.	[70]
Thin-film hydration	Metastatic lung cancer	CXCR4-antagonist cyclic peptide (peptide R)	In vitro cytotoxicity assay. In vivo model: C57BL/6 mice bearing B16 human melanoma cells	N/A	In vitro results showed that targeting significantly decreased the IC_50_ while reducing metastasis and regression in tumor size growth.	[71]
Film dispersion	hepatocellular carcinoma (HCC)	glycyrrhetinic acid (GA) and peanut agglutinin (PNA)	In vitro specific uptake of HepG2, MCF-7, and SMMC-7721 cells In vivo model: male BALB/C-nu mice bearing SMMC-7721 xenografts.	N/A	HepG2 cells showed the highest uptake towards the liposomes functionalized with GA alone, while MCF-7 showed the highest affinity towards the PNA functionalized liposomes. The dual-targeted liposomal formulation was most internalized by the SMMC-7721	[72]

**Table 3:** Phase III clinical trials findings of treatment with Myocet**^®^** and Doxil**^®^** against free DOX in patients with breast cancer [81,82,83].

Formulation	Phase	Therapeutic Indication	Survival Rate (SR)	Progression-Free Survival	Incidence of AEs
			All Presented Comparisons are Against Treatment with Free DOX		
Myocet^®^	III	Metastatic breast cancer	First-year SR: 69% vs. 64%	4.3 vs. 3.6 months	Cardiac events: 13% vs. 29% Mucositis/stomatitis: 8.6% vs. 11.9% Nausea/vomiting: 12.3% vs. 20.3%
DOXIL^®^			Overall SR: 21 months vs. 22 months	6.9 months vs. 7.8 months	Cardiotoxic implications: 3.9% vs. 18.8% Vomiting: 19% vs. 31% Alopecia: 20% vs. 66% Neutropenia: 4% vs. 10% PPE: 48% vs. 2% Stomatitis: 22% vs. 15% Mucositis: 23% vs. 13%

**Table 4:** Summary of preclinical and clinical evaluations of some micellar-based DOX DDS. All presented comparisons are against treatment with free DOX.

Formulation	Composition	Features	Preclinical Studies	Clinical Trials
SP1049C	Pluronic^®^ L61 and L127	Average size~30 nm Physical DOX loading EE~8.2%	In vitro Enhanced activity against multidrug-resistant (MDR) cells In vivo 2-fold higher AUC (14.6 vs. 7.1 μg hr/mL) Lewis lung tumor growth in mice got arrested in more than 50% of 9 tumor models	Phase I: patients with advanced solid tumors Administered doses ranged from 5 to 90 mg/m^2^, once every 3 weeks for six cycles The maximum tolerated dose (MTD) was 70 mg/m^2^ The micellar formulation showed a similar toxicity profile to free DOX 11.5% of the patients had a partial or complete response to the micellar treatment The median time for disease progression: 17.5 weeks in 30.8% of the patients Phase II: patients with advanced adenocarcinoma of the esophagus or gastroesophageal junction Administered dose was 75 mg/m^2^, once every 3 weeks for six cycles Grade 3 or 4 neutropenia was observed in 62% of the patient Median overall survival: 9.96 months Median progression-free survival: 6.6 months Phase III: approved
NK911	poly(ethylene glycol)-b-poly(α,βaspartic acid)	Average size~40 nm DOX was covalently conjugated to 50% of the micelles’ carboxylic groups as well as physically loaded into the cores	In vivo 29-fold higher AUC (120 vs. 4 μg hr/mL) in mice bearing colon-26 carcinoma 3.4-folds higher accumulation at the tumor site (1605 vs. 474 μg hr/mL) effectively arrested the growth of sarcoma, lung, colon and breast cancer in different mouse models	Phase I: patients with metastatic/recurrent solid tumors refractory to conventional DOX chemotherapy Administered doses ranged from 6 to 67 mg/m^2^, once every 3 weeks MTD was 67 mg/m^2^ Grade 3 or 4 neutropenia was observed at doses of 50 mg/m^2^ with AUC of 3.2 vs. 1.6 μg hr/mL The maximum tolerated dose was 70 mg/m^2/^and the recommended dose for phase II trials was 50 mg/m^2^ to be administered once every 3 weeks Phase II: currently undergoing
NC-6300	PEG-p(Asp-Hyd)	Average size~65 nm Modifications to the NK911 formulation by using pH hydrolyzable linkers (hydrazone bonds) for the chemical conjugation of DOX to the micelles	In vivo 15-fold higher AUC (859 vs. 59 μg hr/mL) in mice bearing colon-26 carcinoma 4-folds higher accumulation at the tumor site MTD (40 mg/kg vs. 10 mg/kg)	Phase I: -pending results

**Table 5:** Recent studies on MOF-based DDSs incorporating DOX for cancer treatment.

Composition	Target Cancer	Functionalization	DOX Loading	Study Model	Triggering Modality	Findings	Ref.
nanoscale Zr (IV)-based nanoMOFs (NH2-UiO-66)	hepatocellular carcinoma (HCC)	folic acid (FA), lactobionic acid (LA), glycyrrhetinic acid (GA)	Physical loading at dark conditions for 72 h where 100 mg of each MOFs formulation was added to 35 mg of DOX solution, followed by pelleting and vacuum drying at 40 °C.	Biocompatibility testing by SRB assay on human fibroblast skin cells. In vitro flow cytometry and MTT assay on HepG2 cells.	pH-responsiveness	MOF nanocarriers are biocompatible and safe (cell viability of h 77 ± 0.71% was observed at the highest MOFs concentration of 1000 μg/mL). Drug release from dual-ligand LA-GA formulation was sensitive to pH, releasing 60% and 100% of the encapsulated DOX at pH 7.4 and 4.0, respectively. Dual-targeting was the most efficient approach as these MOFs exhibited the best anti-tumor activity, approaching that of free DOX.	[138]
MIL-100(Al) nanoMOFs	hepatocellular carcinoma (HCC)	γ-cyclodextrincitrate oligomers (CD-CO) coatings	DOX loading was carried out by pelleting the MOFs and dispersing them in water before mixing 1 mL of aqueous MOFs (2 mg/mL) with 1 mL of DOX solution). The mixture was mixed for 1 to 6 days. The loaded MOFs were centrifuged and collected.	Solid-state NMR (ssNMR) spectroscopy. DOX release in phosphate buffer saline (PBS).	N/A	DOX encapsulation efficiency was a function of the weight ratio of DOX to MOFs during the loading process and the time of impregnation. A higher DOX payload was observed with the increase in the weight ratio and the impregnation time. DOX encapsulation had no significant effects on the MOFs’ morphologies or colloidal stability.	[139]
Alendronate (Aln) modified ZIF-8 based MOFs	Bone metastasis	N/A	2 mL of DOX solution (6.8 mg in 50 mL methanol) was mixed with 100 mg of MOFs or Aln-MOFs powder. The mixture was gently mixed for two days, followed by centrifugation, washing, and freeze-drying.	In vitro Cck-8 assay and flow cytometry analysis of mouse breast cancer 4T1 cells. In vivo model: Balb/c mice inoculated with 4T1 cells to establish a bone metastasis model.	pH-responsiveness	DOX entrapment into both types of MOFs resulted in a loaded capacity of 0.65 μg/mg. Release from both types was sustained for 12 h period, while enhanced kinetics were observed at a lower pH (~5.5) than neutral conditions. The modified MOFs (Aln-MOF-DOX) showed superior anti-tumor activity compared to the unmodified MOFs. However, the tumor growth was arrested for 12 days only after which it regrows again.	[140]
Fe-MOFs	Different cancers	cationic polymer MV-PAH multilayers (PEM)	DOX was loaded into Fe-MOFs by mixing 10 mg of DOX with 20 mg of Fe-MOFs overnight, followed by centrifugation. Loaded Fe-MOFs were then coated with PEM using the LBL technique.	The in vitro dialysis bag diffusion technique to study pH-dependent release kinetics, MTT assay to evaluate toxicity to A549 and MCF-7 cells. In vitro Annexin V-FITC apoptosis detection assay.	pH-responsiveness	Both functionalized and unfunctionalized MOFs showed stability and long circulation capabilities. The release at pH 5.0 after 12-h incubation reached 72% in the functionalized MOFs, while unfunctionalized MOFs at pH 7.4 released <4% after the same incubation period. Coating with PEM increased the sensitivity of the DDS towards pH changes.	[141]

## Data Availability

No new data were created or analyzed in this study. Data sharing does not apply to this article.
